# The Clinical Spectrum and Occurrence of Major Infections in Hospitalized Children With Nephrotic Syndrome

**DOI:** 10.7759/cureus.42521

**Published:** 2023-07-26

**Authors:** Waseem Shafi Sheikh, Muzafar Jan, Mohd Ashraf, Aaqib Hamid

**Affiliations:** 1 Pediatrics, Government Medical College Srinagar, Srinagar, IND; 2 Pediatric Nephrology, Government Medical College Srinagar, Srinagar, IND

**Keywords:** pediatric, spontaneous bacterial peritonitis, uti, infections, nephrotic syndrome

## Abstract

Background

Nephrotic syndrome (NS) is one of the most common renal ailments in the pediatric population. The management of NS with major infections remains a challenge to pediatricians and pediatric nephrologists, as it is associated with increased morbidity and mortality. In this study, we aimed to know the clinical spectrum and occurrence of major infections in hospitalized children with NS.

Methods

This prospective, observational study was conducted over a period of two years among hospitalized NS children from one year to 18 years. The clinical spectrum and hospital course were studied in detail, and the data generated were analyzed to obtain valid results.

Results

A total of 101 hospitalizations of 66 children were assessed for the occurrence of infective complications. The incidence rate of infective complications among the hospitalized nephrotics was 29.7%. Urinary tract infection (UTI) was the commonest infective complication, followed by spontaneous bacterial peritonitis (SBP). Other infective complications observed were pneumonia, enteric fever, methicillin-resistant *Staphylococcus aureus* (MRSA) sepsis, tuberculosis, and varicella.

Conclusion

Infective complications are quite common among NS patients, where appropriate identification and prompt treatment could reduce morbidity and mortality.

## Introduction

Nephrotic syndrome (NS) is one of the commonest renal diseases in children. The majority of children with nephrotic syndrome run a steroid-sensitive course with a good long-term prognosis. Children with nephrotic syndrome (NS) have a higher likelihood of developing infections. Although in developed nations, the occurrence of infections in children with NS has reduced, it still remains a significant issue in developing countries [1]. Untreated nephrotic syndrome in children increases the risk of mortality, primarily due to bacterial infections. Prior to the use of corticosteroids and antibiotics, 40% of children died, with 50% of these fatalities resulting from infections [2]. Several significant risk factors for infections include urinary loss of immunoglobulins and alternative complement pathway factors B and I, the presence of edema, and treatment with steroids and other cytotoxic agents [3]. Keeping in view the paucity of data from our region, this study was done to know the clinical spectrum and occurrence of major infections in hospitalized children with NS.

## Materials and methods

This was a prospective, observational study conducted over a period of two years (December 2020 to December 2022) after obtaining approval from the Ethical Committee of Government Medical College Srinagar (GMCS), under the number F(Minutes-BOPGS)Acad/KU/22. All consecutive patients aged between one year and 18 years fulfilling the International Study of Kidney Disease in Children (ISKDC) criteria [4] for the diagnosis of primary nephrotic syndrome, requiring hospitalizations for major complications such as infections, seizures, shock, thrombosis, respiratory distress, or any other complication, were enrolled in the study through general pediatric and pediatric nephrology outpatient department (OPD). Patients with congenital, infantile, or secondary NS and NS patients receiving steroids along with antibiotics were excluded.

Major infections are defined as disseminated, affecting deep organs, requiring hospitalization, or potentially life-threatening [5]. Studies have shown that peritonitis, pneumonia, urinary tract infection (UTI), cellulitis, meningitis, and tuberculosis have been reported as major infections in these children [6-8]. Specific major infections were defined in Table 1.

**Table 1:** Definitions of major infections CSF: cerebrospinal fluid

Major infection	Definition
Peritonitis	Abdominal pain, tenderness, distension, diarrhea, or vomiting, with ascitic fluid >100 leukocytes/mm^3^ and a minimum of 50% neutrophils and/or positive culture [9]
Pneumonia	Lung infection characterized by cough, labored breathing, fever, chest retractions/pain, fatigue, and confusion, with X-ray findings [10]
Urinary tract infection	Bacterial colony count of >10^5^ organisms/mL in a properly collected urine sample with fever (>38.5ºC), dysuria, or increased/difficulty in urination frequency [11]
Cellulitis	Spreading redness of the skin associated with warmth, fever, and affected area that may or may not be painful [12]
Meningitis	Presence of fever, stiff neck, headache, nausea and vomiting, non-blanchable red or purple rash, fatigue or extreme sleepiness, seizures, and irritability or lethargy in infants along with poor feeding, with laboratory documented CSF bacteria/virus/fungal or parasite growth and/or raised white blood cells [13]

Statistical Package for the Social Sciences (SPSS) version 23.0 (IBM SPSS Statistics, Armonk, NY, USA) was used for statistical analysis. P-values of <0.05 were considered statistically significant.

## Results

We studied a total of 66 children who required 101 hospitalizations over a period of two years. The mean age of onset of NS was found to be 6.15 years, with males more commonly involved than females. The incidence of major infections among hospitalized children with nephrotic syndrome was 29.7%, with a female/male ratio of 1.5:1. The mean age of presentation of major infections in NS patients was 6.9 years, while 6-10 years was the commonly involved age group. All (100%) infective patients had fever and anasarca at the time of hospitalization. Urinary tract infection (UTI) was the commonest major infection (53.3%), followed by spontaneous bacterial peritonitis (SBP) (23.3%) and pneumonia (10%). Other infective complications were enteric fever, methicillin-resistant *Staphylococcus aureus* (MRSA) sepsis, tuberculosis, and varicella (3.33% each). Infections such as UTI, pneumonia, SBP, and varicella were more commonly seen among relapsed NS patients. However, enteric fever, MRSA sepsis, and tuberculosis were seen during the first episode of NS. The commonest organism isolated from urine was *Escherichia coli* (43.8%), followed by *Klebsiella* (25%). Hematuria and hypertension were observed in 23.3% of infected NS patients. Other observations are detailed in Table 2 and Table 3.

**Table 2:** Distribution of study population based on the type of infection MRSA: methicillin-resistant *Staphylococcus aureus*

Major infection	Number of patients	Percentage
Enteric fever	1	3.33
MRSA sepsis	1	3.33
Pneumonia	3	10
Spontaneous bacterial peritonitis	7	23.34
Tuberculosis	1	3.33
Urinary tract infection	16	53.34
Varicella	1	3.33
Total	30	100

**Table 3:** Common organisms causing urinary tract infections among hospitalized nephrotic syndrome patients

Organism	Number of patients	Percentage
Escherichia coli	7	43.8
Klebsiella	4	25
Enterococcus	3	18.7
Proteus	2	12.5
Total	16	100

## Discussion

Bacterial infections are common among NS patients and carry substantial morbidity and mortality. The mean age of children with major infections in NS patients was found to be 6.9 years, which was in concordance with the findings of Gulati et al. [6], Kumar et al. [14], and Lebel et al. [15]. Infective complications were found more in females than in males (1.5:1), which is in concordance with the results of Rahman et al. [16]. The preponderance of females having more infective complications is probably due to the fact that females are at high risk of developing UTI than males, since we observed that UTI was the commonest type of infective complication. However, Kumar et al. [14] and Krishnan et al. [17] showed male predominance in NS patients with infective complications.

In our study, the incidence of major infection in hospitalized children with nephrotic syndrome was 29.7%, which is in concordance with the findings of Senguttuvan et al. [18], who reported an incidence of 31%. Ajayan et al. [8] reported a 36.6% incidence of major infections in hospitalized children with NS.

The commonest infection in our study was urinary tract infection, accounting for 53.3% of total major infections. This is in confirmation with the study by Senguttuvan et al. [18] and Gulati et al. [19]. However, studies done by Ajayan et al. [8] and Kumar et al. [14] reported SBP as the commonest infection among hospitalized NS patients. The majority of these UTI patients presented to us with complaints of fever, abdominal pain, and burning micturition. *Escherichia coli* was the commonest pathogen isolated in the urine culture of these patients, followed by *Klebsiella*, which was in concordance with the findings of Senguttuvan et al. [18] and Gulati et al. [19]. These patients responded well to third-generation cephalosporins. It is worth mentioning here that 43.7% of UTIs occurred during the first episode of NS.

SBP was the second most common infection seen in hospitalized NS patients, accounting for 23.3% of major infections. This is quite similar to the study by Senguttuvan et al., where they reported peritonitis in 25.8% of total NS infections [18]. However, Ajayan et al. [8] reported 37.8% of SBP cases among total major infections. From our study, it came forth that SBP was commonly seen more in NS relapse (57.1%), an observation well in confirmation with the findings of Krishnan et al. [17]. These patients had a fever, abdominal pain, generalized edema, loose stools, and vomiting as common clinical presentations.

We observed lower respiratory tract infection (pneumonia) as the third most common infection (10%) among hospitalized children with nephrotic syndrome. This is in concordance with the study done by Ajayan et al. [8], who reported 12.9% of cases of pneumonia. In contrast, Krishnan et al. [17] reported 41.7% of pneumonia cases as the commonest infective complication. These patients presented with complaints of fever, cough, and fast breathing along with chest X-ray findings.

In the present study, one (3.33%) patient had tuberculosis, which is similar to the observations made by Kumar et al. [14] and Krishnan et al. [17], reporting the prevalence of tuberculosis from 2.1% to 2.8% among infective nephrotics. However, Gulati et al. [1] reported a prevalence of 10.4% of tuberculosis among NS patients. This could be because of the high endemicity of tuberculosis in that geographical area.

Although few studies have reported septicemia as more common among NS patients, we had one (3.33%) patient with septicemia, which is congruent with the findings of Easin et al. [20], who reported septicemia in 3.3% of NS patients, which is contrary with the findings of Krishnan et al. [17], where 16.7% of NS patients had septicemia, which could possibly be due to stronger immune suppression and associated malnutrition.

Enteric fever was diagnosed in one (3.33%) patient, which is similar to earlier studies [14,18]. Pertaining to viral infections, the present study shows 3.33% of varicella among major infections in hospitalized nephrotic syndrome patients, which is in line with the observation made by Ajayan et al. [8], who also reported 2.7% of varicella in their study. We had zero mortality due to prompt recognition and timely management of these patients.

This study had some limitations. Being a single-center study with low power, these findings warrant cautious interpretation. The occurrence rate of NS with major infections could be more than our findings as many patients might have opted for other hospitals for treatment.

## Conclusions

More than 29% of NS patients were hospitalized with underlying major infective pathology, among whom UTI was the commonest infection, followed by SBP. Other major infections observed were pneumonia, enteric fever, MRSA sepsis, tuberculosis, and varicella. The commonest organism causing UTI was *Escherichia coli*, followed by *Klebsiella*. Prompt and appropriate treatment in such situations reduces mortality and morbidity.
